# Diagnostic approach with *Z*-score mapping to reduce artifacts caused by cerebral atrophy in regional CBF assessment of mild cognitive impairment (MCI) and Alzheimer's disease by [^99m^Tc]-ECD and SPECT

**DOI:** 10.1007/s11604-023-01526-8

**Published:** 2024-02-14

**Authors:** Ikuo Odano, Fumio Maeyatsu, Tetsuo Hosoya, Mami Asari, Kentaro Oba, Yasuyuki Taki

**Affiliations:** 1 Department of Aging Research and Geriatric Medicine, Institute of Development, Aging and Cancer, Tohoku University, Sendai, Japan; 2 Department of Neurology and Radiology, Miyagi Kosei Association, Izumi Hospital, Sendai, Japan; 3 Department of Software Development. Division of Quality, Safety Management and Regulatory Affairs, PDRadiopharma. Inc., Tokyo, Japan; 4 Department of Radiology and Radiation Oncology, Niigata University Graduate School of Medicine and Dental Sciences, Niigata, Japan; 5 Department of Human Brain Science, Institute of Development, Aging and Cancer, Tohoku University, Sendai, Japan; 6 Department of Aging Research and Geriatric Medicine, Institute of Development, Aging and Cancer, Tohoku University, 4-1 Seiryo-cho, Aoba-ku, Sendai, 980-8575 Japan

**Keywords:** Mild cognitive impairment, Alzheimer's disease, Cerebral blood flow, Brain atrophy, SPECT, SPM

## Abstract

**Purpose:**

The aim of this study was to develop a novel approach that enhanced diagnostic accuracy in the diagnosis of mild cognitive impairment (MCI) and early Alzheimer's disease (AD) using cerebral perfusion SPECT by minimizing artifacts caused by cerebral atrophy.

**Materials and methods:**

[^99m^Tc]-ECD and SPECT studies were performed on 15 cognitively normal patients, 40 patients with MCI, and 16 patients with AD. SPECT images were compared using SPM. The atrophy correction method was incorporated to reduce artifacts through the MRI masking procedure. Regional *Z*-score, percent extent, and atrophy correction rate were obtained and compared. The *Z*-score mapping program was structured as a single package that ran semi-automatically.

**Results:**

The method significantly reduced regional *Z*-score in most regions, leading to improved estimates. The mean atrophy correction rate ranged from 10.4 to 12.0%. In MCI and AD, the convexities of the frontal and parietal lobes and the posterior medial cerebrum were particularly sensitive to cerebral atrophy, and the *Z*-scores were overestimated, whereas the posterior cingulate cortex and the cerebellum were less sensitive. The diagnostic accuracy for MCI increased from 67 to 69% and for AD from 78 to 82%.

**Conclusion:**

The proposed approach provided more precise *Z*-scores with less over- or underestimation, artifacts, and improved diagnostic accuracy, being recommended for clinical studies.

## Introduction

Diagnosis of mild cognitive impairment (MCI) and early-stage dementia in Alzheimer's disease (AD) among the elderly is a pressing challenge in medicine. MCI is defined as the boundary between memory loss and impairment, and is regarded as a preclinical stage of dementia, with an average rate of progression to dementia of 5–15% each year [1–4]. AD is the most common form of dementia, and the condition of MCI requires preclinical and non-medicinal interventions to prevent further cognitive decline [5]. Previously, we reported that whole-body vibration exercises enhance cognitive decline in patients with MCI [6]. Also, the development of disease-modifying therapies for early AD is rapidly progressing [7]. Therefore, it is increasingly important to accurately evaluate MCI and early AD.

The measurement of regional cerebral blood flow (rCBF) by single photon emission computed tomography (SPECT) is a key technique in the workup of MCI, AD, and other neurodegenerative dementia. Tracers of [^99m^Tc] ethylene cysteinate dimer (ECD), as well as N-isopropyl-p [^123^I] iodoamphetamine (IMP) are widely used in evaluating rCBF in clinical studies. The former is evaluated with software incorporating statistical parametric mapping (SPM) [8], and the latter with 3D-SSP and voxel-based analysis [9], each of which allows semi-automatic analysis to assess rCBF changes.

On the other hand, neurodegenerative disorders often lead to various extents of brain shrinkage [10], with cerebral atrophy also reported in elderly people with subjective memory complaints [11]. Moreover, cerebral atrophy and ventricle enlargement are both associated with the normal aging process [12]. Such age- and disease-related cerebral morphology, i.e., brain atrophy, enlarged ventricles and cisterns, as well as congenital or acquired brain tissue defects, produce artifacts in SPM analyses leading to overestimated rCBF reductions [13]. These artifacts complicate the identification of regions of true hypoperfusion [4], reducing diagnostic accuracy, hence some solution has been needed that is used in routine clinical work.

The purpose of the present study was to propose a novel diagnostic approach for SPECT with [^99m^Tc]-ECD to reduce artifacts and appropriately assess *Z*-scores that better reflect rCBF. This approach employed an atrophy correction method that minimizes artifacts as well as over- or underestimation caused by brain morphology changes. We investigated which regions were more susceptible to artifacts in the SPM analysis caused by cerebral atrophy, and how the proposed method thus improved diagnostic accuracy. Moreover, specific attention was given to five dementia-related regions and attempts were made to recommend routine clinical studies. Henceforth, in this study, the various brain morphology changes were represented by a single term, cerebral atrophy.

## Materials and methods

### Theory of Z-score mapping approach (*Zsmap*) and atrophy correction method

**Fig. 1:**
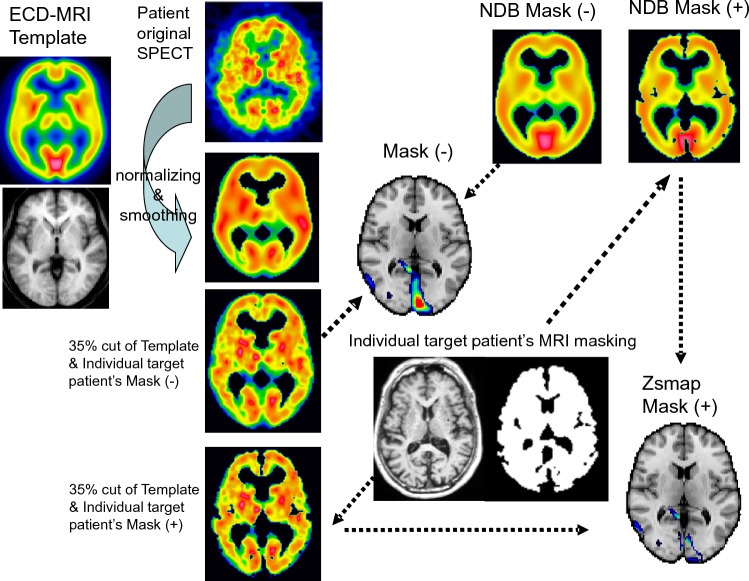
An outline of the Z‑score mapping approach reducing artifacts caused by cerebral atrophy, named *Zsmap*

This prospective study was approved by the institutional review board and performed after obtaining informed consent. An outline of the semi-automatically operated approach is shown in Fig. 1. To create a stereotactic space template, both axial [^99m^Tc]-ECD SPECT images, MR-T1W images and brain CT images of five normal subjects were transformed into the same stereotactic space using SPM2 [14] and MATlab (Math Works, Natick, MA, USA). SPM2 was used as it is robust and easy to use. As MATlab was integrated into the program, it was no longer needed. The [^99m^Tc]-ECD SPECT images were smoothed by convolution using a 12 mm full width at half maximum (FWHM) isotropic Gaussian kernel. An average image was obtained from the five [^99m^Tc]-ECD SPECT images, and the left side of the average image folded to the right, yielding a symmetrical [^99m^Tc]-ECD SPECT template. This symmetrical template allows evaluation of right-left differences in the target SPECT images. Then, a patient’s [^99m^Tc]-ECD SPECT images were standardized and smoothed using the symmetrical template, followed by masking by the symmetrical template with a threshold set at 35% of the maximum count [8].

To analyze and statistically compare patient SPECT images with age-matched control subjects, the threshold of 35% of the maximum count was set, and voxel-by-voxel *Z*-scores were calculated using the following equation: *Z*-score = ([control means] − [individual subject mean])/(control standard deviation) with analysis after voxel normalization to the global mean. Then, the obtained *Z*-scores were displayed in color scale on a three-dimensional topography section of the anatomically standardized MRI template [15] based on the orbito-meatal line mentioned later.

To reduce image processing artifacts caused by cerebral atrophy, an atrophy correction method was incorporated with the masking procedure of the patient's MRI. Using the patient's MRI mask, extra cerebral space was removed from SPECT images of both patients and comparative age-matched controls. One-mm thickness MR-T1WI of the patient was segmented into gray matter, white matter and CSF space using SPM2. The images were smoothed with an isomeric 4-mm FWHM Gaussian kernel, and binary volume images created from those original images. The binary images of white matter were added to those of gray matter, thus creating mask images of the patient. These mask images were used to eliminate voxels outside brain tissue from the reconstructed SPECT images of the target patient, as well as from the SPECT images of age-matched control subjects. Then voxel-by-voxel comparison was performed and displayed on the anatomically standardized MRI template with *Z*-scores. Hereafter, the case of analysis using the individual mask processing is represented by Mask (+), and the conventional case of analysis without individual mask processing by Mask (−).

In the present SPECT studies, patient data were obtained with a dual-detector gamma camera (Siemens ECAM in Izumi Hospital, Sendai, Japan) described later, while data for age-matched control subjects were obtained with the triple-detector gamma camera (Toshiba GCA9300 A/HG in Niigata University Medical and Dental Hospital, Niigata, Japan) [16]. The data from control subjects were used with the approval of Niigata University Medical and Dental Hospital, as they were generated at different institutions. Furthermore, SPECT data of control subjects were standardized for individual radioactivity, and only mean values and standard deviations were used, without any personal information. To compare both data sets, the patient's SPECT data obtained with the dual-detector gamma camera were transformed using the phantom correction method [17], the program of which was provided by PDRadiopharma Inc., Japan, then compared with SPECT data of the age-matched control subjects.

The orbito-meatal (OM) line was newly introduced as the anatomical standardized image reference line replacing the anterior commissure- posterior commissure (AC-PC) line. Specifically, the difference in angle between the AC-PC line and the OM line was analyzed after superimposing the respective axial [^99m^Tc]-ECD SPECT, brain MR-T1W images and brain CT images of the five normal subjects described above. Consequently, the difference in angle was determined to be an average (SD) of 10.0 (4.0) degrees. Then the 3D standardized MRI template images were rotated 10.0 degrees counterclockwise to be parallel to the OM line. *Z *-scores were similarly transformed and displayed on the template using a color scale.

In addition, to obtain the hippocampal topographic section, the standardized MRI template was rotated approximately 34.0 degrees counterclockwise from the AC-PC plane, the angle deemed appropriate by inspection, and the *Z*-scores obtained displayed on it. All the above programs were assembled as one package, operated semi-automatically, and referred to as* Zsmap*.

### Participants

Fifteen patients diagnosed in the cognitively normal range, i.e., cognitively normal patients, 40 patients with MCI, and 16 patients with probable AD participated in this study (Table 1). They were recruited from patients who visited Miyagi Kosei Association, Izumi Hospital (Sendai, Japan) between December 2016 and February 2022 with subjective or objective memory loss as their main complaint. Multiple domains were excluded for patients with MCI, and included for patients with amnestic MCI, hereinafter collectively referred to simply as MCI. All patients underwent somatic and neurological examinations, neuropsychological tests including the Mini-Mental State Examination (MMSE) and Montreal Cognitive Assessment Japanese version (MoCA-J), along with laboratory examinations, [^99m^Tc]-ECD SPECT, and cerebral MR studies. A clinical diagnosis was established according to the criteria of MCI [18, 19], and probable AD without evidence of the AD pathophysiological process [20]. Patient selection was also performed according to the criteria by neuropsychological tests: 25 ≤ MMSE ≤ 30 and 27 ≤ MoCA-J ≤ 30 for cognitively normal, 22 ≤ MMSE < 28 and 21 ≤ MoCA-J  ≤ 26 for MCI, and MMSE ≤ 21 and MoCA-J ≤ 20 for probable AD. When being difficult to judge the three groups, priority was given to MoCA-J. [^99m^Tc]-ECD SPECT findings were not taken into account in the process of patient group classification. MR imaging revealed neither cerebral infarction nor bleeding, and patients with moderate or severe ischemic white matter lesions were excluded.

**Table 1:** Participant characteristics

	Cognitively normal	MCI	AD
Number (man)	15 (8)	40 (25)	16 (10)
Age, y	70 (9.0)ns	71 (7.7)ns	74 (8.0)ns
MMSE	29 (1.2)**	27 (1.8)**	17. (5.5)**
MoCA-J	27 (1.0)**	22 (2.4)**	12 (5.8)**

*MCI* mild cognitive impairment; *AD* Alzheimer’s disease; *MMSE* Mini-Mental State Examination score; *MoCA-J* Montreal Cognitive Assessment Japanese version; *ns*: not significant

**p < 0.01

### Radiotracer and SPECT data acquisition

A dose of 600 MBq of [^99m^Tc]-ECD (PDRadiopharma Inc. Japan) was injected with eyes closed. Ten minutes after tracer injection, SPECT data were acquired using a dual-detector gamma camera (Siemens ECAM, Japan) equipped with low-medium energy fanbeam (LMEFAN) collimators. The data obtained were processed as previously reported [6].

### MR images

Brain MR images of each participant were acquired using a 1.5 Tesla MAGNETOM Symphony (Siemens Healthcare Japan) with sagittal three-dimensional T1_MPRAGE [TR/T1/TE = 2400/1000/3.8 ms, flip angle 8.0°, FOV 24 cm, 256 × 256 matrix, 1.2-mm thickness, gapless] were obtained. The sagittal T1WIs were re-sliced into axial images with a thickness of 1.0 mm, and used to create individual masks.

### Data analysis for comparisons and atrophy correction rate

By applying the vbSEE method (Levels 1–5) [21], 145 regions of interest (ROIs) were defined on both Mask (−) and Mask (+) images of SPECT, with *Z*-scores and extent values (%) obtained and compared. Of all ROIs, 24 ROIs with a *Z*-score greater than 0.5 on the left- or right-side cerebrum were selected, the value of which was at least pathological, then the mean of the left and right *Z *-scores, and extent (%) obtained.

To compare the *Z*-score for Mask (−) and Mask (+), the atrophy correction rate (%) was defined as follows:

1 $$\begin{aligned} {\text{atrophy}}\;{\text{correction}}\;{\text{rate}}& = \left[ {Z_{{{\text{score}}}} {\text{Mask}}\left( - \right) - Z_{{{\text{score}}}} {\text{Mask}}( + )} \right]\\& \quad /Z_{{{\text{score}}}} {\text{Mask}}\left( - \right)*100. \end{aligned}$$

When the atrophy correction rate was positive, the *Z*-score Mask (−) overestimated, and if negative, underestimated. The atrophy correction rates for the extent (%) for Mask (−) and Mask (+) were also obtained using Eq. (1), and compared.

### Five dementia-related regions

Since it is crucial to evaluate decreased rCBF in the posterior regions of the cerebrum in the diagnosis of dementia, five dementia-related regions were selected: the superior parietal lobule, angular gyrus, supramarginal gyrus, precuneus, and the posterior cingulate cortex. The *Z*-scores were compared. The receiver operating characteristic (ROC) curve analysis was performed, obtaining the optimal cutoff point using Youden's index.

### Statistics

Differences in patient characteristics were evaluated using one-way ANOVA with the Steel–Dwass test and chi-squared test for categorical variables. Significant differences in *Z *-scores and extent values between Mask (−) and Mask (+) were assessed using the paired Student’s *t* test on two sides. Significant differences between the three groups were assessed by one-way ANOVA using the Tukey–Kramer procedures, ROC curve analysis, and $\chi^{2}$ for independent test. Significance was determined at *p* < 0.05.

## Results

The Mask (+) procedure significantly reduced *Z*-scores with mean atrophy correction rates (SD) of 12.0 (11.1) % for cognitively normal, 10.5 (10.5) % for MCI and 10.4 (9.8) % for AD, (*p* < 0.001), as listed in Tables 2, 3 and 4. The regions where *Z*-scores were not reduced by Mask (+) were the cerebellum in cognitively normal and MCI, and the superior and middle occipital gyri in AD.

**Table 2:** Comparisons of *Z*-scores with and without masking procedures and atrophy correction rate at each ROI for cognitively normal patients (*n* = 15)

Cognitively normal	Mask (−)	Mask (+)	*p* value	Atrophy correction rate (%)	CV
Frontal lobe					
Superior frontal gyrus	0.77 (0.21)	0.69 (0.19)	0.004**	9.8 (10.1)	1.03
Medial frontal gyrus	0.71 (0.25)	0.63 (0.23)	< 0.001**	11.7 (8.4)	0.71
Inferior frontal gyrus	0.66 (0.23)	0.54 (0.16)	0.001**	17.1 (12.3)	0.72
Paracentral lobule	0.84 (0.27)	0.79 (0.28)	0.010**	6.8 (8.5)	1.24
Parietal lobe					
Superior parietal lobule	1.12 (0.30)	0.97 (0.26)	< 0.001**	12.6 (9.3)	0.74
Angular gyrus	1.12 (0.34)	0.93 (0.32)	0.001**	17.4 (14.8)	0.85
Supramarginal gyrus	0.99 (0.27)	0.80 (0.24)	< 0.001**	19.1 (16.2)	0.85
Precuneus	1.03 (0.28)	0.89 (0.22)	0.002**	11.8 (10.0)	0.85
Postcentral gyrus	0.92 (0.26)	0.81 (0.21)	< 0.001**	10.9 (7.6)	0.69
Temporal lobe					
Superior temporal gyrus	0.75 (0.14)	0.67 (0.15)	0.009**	11.0 (13.0)	1.18
Middle temporal gyrus	0.88 (0.17)	0.77 (0.18)	< 0.001**	12.5 (10.7)	0.86
Inferior temporal gyrus	0.81 (0.17)	0.72 (0.17)	< 0.001**	11.4 (7.4)	0.65
Occipital lobe					
Superior occipital gyrus	1.20 (0.41)	0.94 (0.35)	< 0.001**	20.2 (15.7)	0.78
Middle occipital gyrus	1.04 (0.37)	0.91 (0.36)	< 0.001**	13.5 (7.8)	0.57
Cuneus	1.05 (0.23)	0.93 (0.22)	< 0.001**	11.1 (8.8)	0.80
Lingual gyrus	0.66 (0.29)	0.60 (0.28)	0.012*	10.5 (12.1)	1.16
Limbic lobe					
Cingulate cortex	0.83 (0.18)	0.70 (0.15)	< 0.001**	14.5 (9.3)	0.64
Posterior cingulate cortex	0.78 (0.29)	0.70 (0.24)	0.014*	9.1 (9.1)	1.00
Retrosplenial cortex (BA26, 29,30)	0.73 (0.31)	0.63 (0.24)	0.004**	13.3 (7.9)	0.60
Hippocampus	0.40 (0.23)	0.32 (0.22)	< 0.001**	26.6 (19.4)	0.73
Cerebrum	0.89 (0.11)	0.80 (0.09)	< 0.001**	9.8 (6.5)	0.66
Cerebellum	1.12 (0.40)	1.11 (0.46)	0.973ns	1.7 (17.7)	10.13
Gray matter	0.89 (0.12)	0.80 (0.09)	< 0.001**	9.9 (6.6)	0.67
White matter	0.89 (0.11)	0.80 (0.10)	< 0.001**	10.0 (6.2)	0.63
Mean	0.78 (0.33)	0.68 (0.30)	< 0.001**	12.0 (11.1)	0.92
Five dementia-related regions	1.01 (0.31)	0.86 (0.27)	0.015*	14.1 (12.5)	0.89

Mean (SD), atrophy correction rate (%) and its coefficient of variation (CV), and *p* values are shown. Five dementia- related regions: the superior parietal lobule, the angular gyrus, the supramarginal gyrus, the precuneus and the posterior cingulate cortex. Mask (−): without the masking procedure. Mask (+): with the masking procedure

*BA* Brodmann area; *ns* not significant

*p < 0.05, **p < 0.01

**Table 3:** Comparisons of *Z*-scores with and without masking procedures and atrophy correction rate at each ROI for patients with MCI (*n* = 40)

MCI	Mask (−)	Mask (+)	*p* value	Atrophy correction rate (%)	CV
Frontal lobe					
Superior frontal gyrus	0.92 (0.26)	0.77 (0.26)	< 0.001**	16.6 (12.5)	0.76
Medial frontal gyrus	0.99 (0.30)	0.88 (0.28)	< 0.001**	11.5 (10.4)	0.91
Inferior frontal gyrus	0.61 (0.23)	0.51 (0.18)	< 0.001**	14.6 (12.8)	0.88
Paracentral lobule	1.48 (0.67)	1.38 (0.63)	< 0.001**	8.0 (7.0)	0.87
Parietal lobe					
Superior parietal lobule	1.97 (0.75)	1.73 (0.65)	< 0.001**	12.5 (4.4)	0.35
Angular gyrus	1.25 (0.48)	1.09 (0.43)	< 0.001**	12.4 (5.5)	0.44
Supramarginal gyrus	1.13 (0.38)	1.02 (0.35)	< 0.001**	10.0 (7.0)	0.69
Precuneus	1.76 (0.58)	1.55 (0.49)	< 0.001**	11.5 (4.3)	0.37
Postcentral gyrus	1.27 (0.44)	1.09 (0.37)	< 0.001**	13.2 (6.6)	0.50
Temporal lobe					
Superior temporal gyrus	0.81 (0.23)	0.74 (0.22)	< 0.001**	7.7 (10.5)	1.37
Middle temporal gyrus	0.93 (0.23)	0.87 (0.23)	< 0.001**	6.8 (4.5)	0.67
Inferior temporal gyrus	0.56 (0.21)	0.51 (0.20)	< 0.001**	9.0 (9.0)	0.99
Occipital					
Superior occipital gyrus	1.27 (0.52)	1.10 (0.45)	< 0.001**	13.7 (6.3)	0.46
Middle occipital gyrus	1.41 (0.40)	1.30 (0.38)	< 0.001**	7.5 (5.2)	0.69
Cuneus	1.40 (0.51)	1.25 (0.44)	< 0.001**	1.07 (4.0)	0.37
Lingual gyrus	0.98 (0.50)	0.86 (0.32)	< 0.001**	9.6 (8.3)	0.86
Limbic lobe					
Cingulate cortex	1.25 (0.39)	1.12 (0.36)	< 0.001**	11.2 (7.1)	0.63
Posterior cingulate cortex	1.68 (0.70)	1.60 (0.69)	< 0.001**	5.9 (7.2)	1.21
Retrosplenial cortex (BA26, 29,30)	1.38 (0.63)	1.28 (0.56)	< 0.001**	7.0 (10.0)	1.39
Hippocampus	0.41 (0.29)	0.35 (0.26)	< 0.001**	19.9 (27.6)	1.39
Cerebrum	1.21 (0.25)	1.10 (0.21)	< 0.001**	1.6 (8.0)	4.99
Cerebellum	1.22 (0.34)	1.20 (0.32)	0.248ns	8.8 (4.6)	0.52
Gray matter	1.23 (0.28)	1.11 (0.23)	< 0.001**	9.9 (4.6)	0.47
White matter	1.17 (0.20)	1.08 (0.19)	< 0.001**	7.8 (3.8)	0.49
Mean	1.23 (0.20)	1.06 (0.51)	< 0.001**	10.5 (10.5)	1.00
Five dementia-related regions	1.56 (0.67)	1.40 (0.61)	< 0.001**	10.3 (6.2)	0.59

Mean (SD), atrophy correction rate (%) and its coefficient of variation (CV), and *p* values are shown. Five dementia-related regions: the superior parietal lobule, the angular gyrus, the supramarginal gyrus, the precuneus and the posterior cingulate cortex. Mask (−): without the masking procedure. Mask (+): with the masking procedure

*BA* Brodmann area; *ns* not significant

*p < 0.05, **p < 0.01

**Table 4:** Comparisons of *Z*-scores with and without masking procedures and atrophy correction rate at each ROI for patients with AD (n = 16)

AD	Mask (−)	Mask (+)	*p* value	Atrophy correction rate (%)	CV
Frontal lobe					
Superior frontal gyrus	1.10 (0.57)	0.88 (0.52)	0.001**	21.3 (12.2)	0.57
Medial frontal gyrus	0.81 (0.40)	0.69)0.38)	< 0.001**	15.3 (12.7)	0.83
Inferior frontal gyrus	1.01 (0.37)	0.87 (0.31)	< 0.001**	13.3 (10.3)	0.77
Paracentral lobule	1.11 (0.46)	0.98 (0.43)	< 0.001**	12.8 (8.9)	0.70
Parietal lobe					
Superior parietal lobule	1.73 (0.47)	1.48 (0.43)	< 0.001**	14.6 (7.8)	0.53
Angular gyrus	1.98 (0.74)	1.80 (0.76)	< 0.001**	10.3 (5.5)	0.53
Supramarginal gyrus	1.57 (0.63)	1.43 (0.60)	< 0.001**	9.6 (4.4)	0.46
Precuneus	1.56 (0.37)	1.37 (0.35)	< 0.001**	12.1 (6.0)	0.50
Postcentral gyrus	0.86 (0.31)	0.68 (0.29)	< 0.001**	22.3 (15.1)	0.68
Temporal lobe					
Superior temporal gyrus	1.06 (0.32)	1.05 (0.42)	< 0.001**	2.6 (14.3)	5.54
Middle temporal gyrus	1.40 (0.54)	1.35 (0.62)	< 0.001**	4.6 (8.1)	1.76
Inferior temporal gyrus	0.98 (0.55)	0.86 (0.47)	< 0.001**	12.1 (5.6)	0.46
Occipital lobe					
Superior occipital gyrus	1.45 (0.73)	1.27 (0.75)	0.818 ns	15.4 (8.2)	0.53
Middle occipital gyrus	1.49 (0.55)	1.40 (0.63)	0.272ns	7.5 (6.7)	0.89
Cuneus	1.27 (0.35)	1.16 (0.36)	< 0.001**	9.4 (6.1)	0.65
Lingual gyrus	0.80 (0.33)	0.74 (0.30)	0.001**	7.8 (9.0)	1.14
Limbic lobe					
Cingulate cortex	1.47 (0.36)	1.35 (0.34)	< 0.001**	7.6 (4.2)	0.54
Posterior cingulate cortex	1.59 (0.59)	1.51 (0.58)	0.025*	5.9 (5.8)	0.98
Retrosplenial cortex(BA26, 29,30)	1.24 (0.68)	1.14 (0.61)	< 0.001**	8.8 (8.9)	1.02
Hippocampus	1.00 (0.54)	0.81 (0.46)	< 0.001**	20.2 (11.6)	0.58
Cerebrum	1.29 (0.21)	1.19 (0.23)	0.003**	7.8 (6.4)	0.83
Cerebellum	0.83 (0.35)	0.76 (0.33)	< 0.001**	8.2 (5.9)	0.72
Gray matter	1.27 (0.20)	1.16 (0.21)	0.005**	8.5 (6.4)	0.75
White matter	1.30 (023)	1.20 (0.26)	< 0.001**	7.9 (6.2)	0.79
Mean	1.26 (0.55)	1.13 (0.54)	< 0.001**	10.4 (9.8)	0.94
Five dementia-related regions	1.69 (0.58)	1.52 (0.57)	< 0.001**	10.6 (6.5)	0.62

Mean (SD), atrophy correction rate (%) and its coefficient of variation (CV), and *p* values are shown. Five dementia-related regions: the superior parietal lobule, the angular gyrus, the supramarginal gyrus, the precuneus and the posterior cingulate cortex. Mask (−): without the masking procedure. Mask (+): with the masking procedure

*BA*: Brodmann area; *ns*: not significant

*p < 0.05, **p < 0.01

**Fig. 2:**
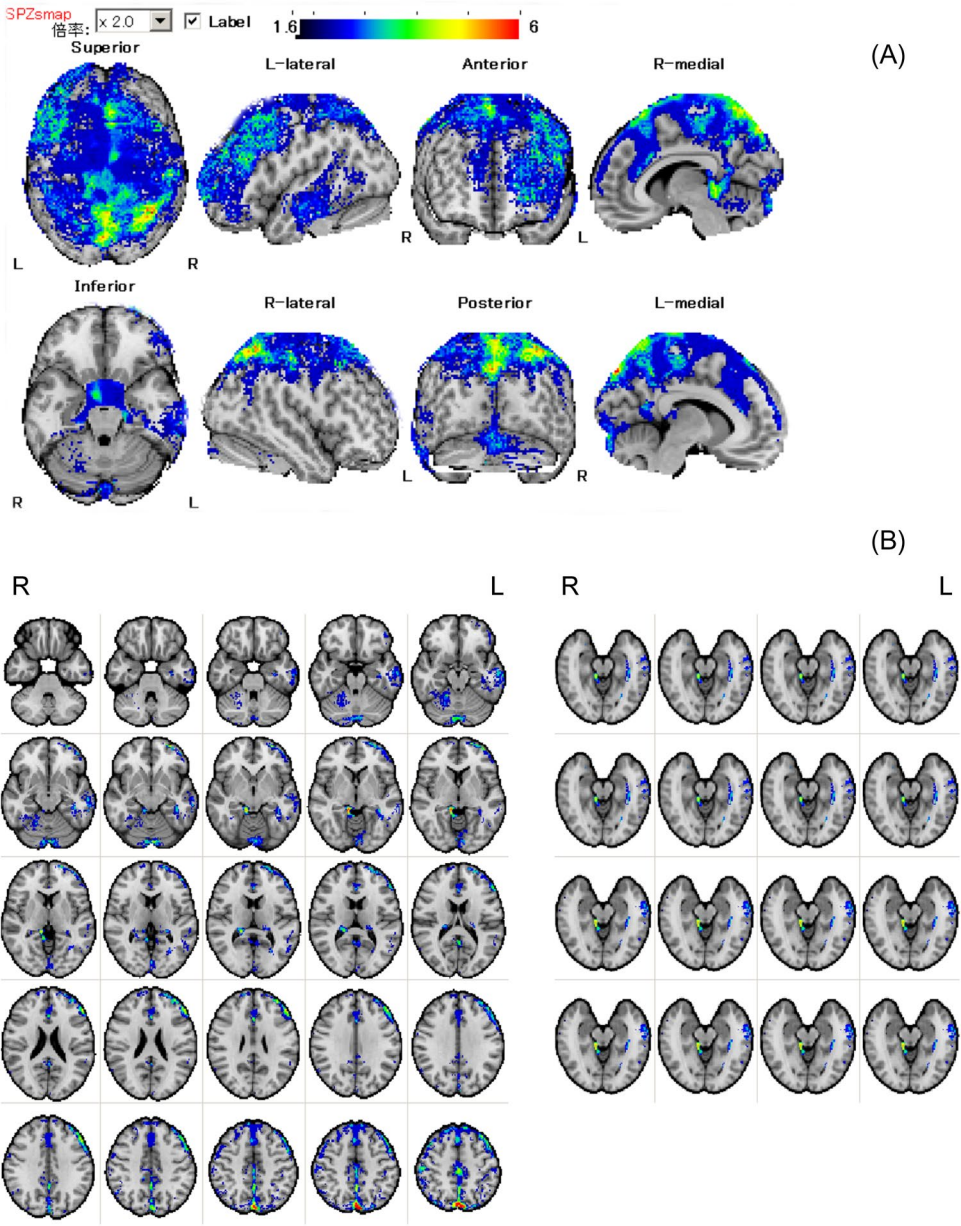
Images [mean Mask (−) + SD] − [mean Mask (+) + SD] of patients with MCI and AD (*n* = 56) are shown. Color image areas displayed on the standardized MRI parallel to the orbital–orbital line are regions of *Z*-score overestimation caused by cerebral atrophy, in the surface images (**A**), axial and hippocampal slice images (**B**). Notably, the convexities of the frontal and parietal lobes, the posterior medial part of the cerebrum and periarcuate brain tissue of ventricles and cisternae are prominently affected by cerebral atrophy

**Fig. 3:**
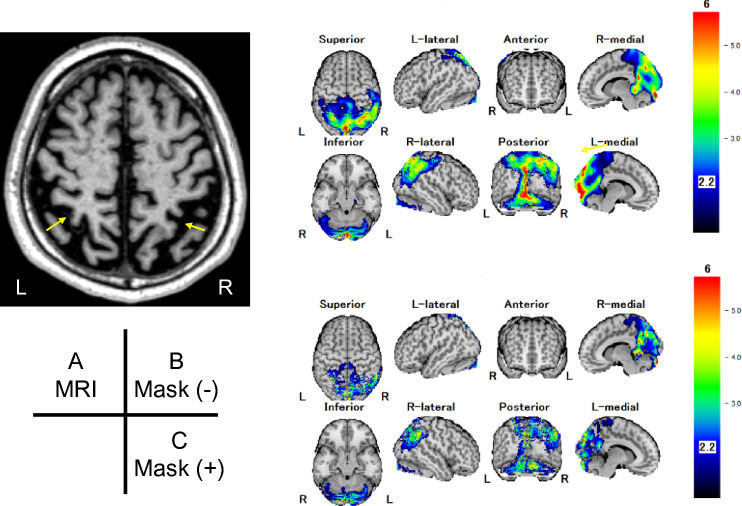
A 70-year-old man with MCI. MRI revealed widening of bilateral intraparietal sulci and the longitudinal cerebral fissure, i.e., moderate to severe cerebral atrophy in the parietal lobes (A-arrows). The Mask (−) images showed extensive high *Z*-scores in the bilateral parietal lobes, the precuneus, and the medial occipital lobes (B), the finding of which could be diagnosed as Alzheimer's disease dementia or dementia with Lewy bodies (DLB) complications. In contrast, the mask(+) images (C) showed less artifacts and lower *Z*-scores, which would be diagnosed as suspected MCI due to AD or early AD. In fact, the patient's clinical symptoms were mild and did not progress over the 6-year follow-up without any treatment, suggesting that Mask (+) more adequately reflected the disease than Mask (−)

To investigate the reduction in *Z*-scores for MCI and AD (56 patients in total), [mean Mask (−) + SD] − [mean Mask (+) + SD] images were created, as shown in Fig. 2. The *Z*-scores were markedly reduced in the dorsolateral and medial prefrontal cortex, convexity of the frontal lobe, temporal lobe, parietal lobe, cingulate cortex, precuneus, and brain tissue surrounding the longitudinal cerebral fissure and ventricles. Remarkable reductions were observed around various cisternae, particularly the pericallosal cistern, the chiasmatic cistern, the interpedincular cistern, the ambient cistern and the quadrigeminal cistern. Example images of a 70-year-old man with MCI are shown in Fig. 3.

Comparing the percent extent values for Mask (−) and Mask (+) in the three groups, the mean atrophy correction rates of extent (%) Mask (−) were significantly reduced with a mean value of more than 24% in each group (*p* < 0.001).

### Five dementia-related regions

The mean *Z*-scores and atrophy correction rates for the five dementia-related regions are summarized and shown in Table 5. The average *Z*-scores were significantly reduced by Mask (+) in each region of the three groups (*p* < 0.01). Focusing on Mask(+), multiple comparison tests of the *Z*-score of the three groups showed that in cognitively normal vs. MCI, the *Z*-score and their averages in the superior parietal lobule, precuneus, and posterior cingulate cortex of the MCI group were significantly higher (*p* < 0.0001). In contrast, cognitively normal vs. AD had significantly higher *Z*-scores and their averages in all regions in the AD group (*p* < 0.05–*p* < 0.001). Furthermore, MCI vs. AD multiple comparison tests showed significantly higher *Z*-scores for the angular gyrus and supramarginal gyrus (*p* < 0.0001 and *p* < 0.01, respectively) in the AD group, however, there were no differences in other regions or averages. In other words, in MCI, the *Z*-score of the angular gyrus and supramarginal gyrus was comparable to that of cognitively normal, while in AD it was significantly higher, and the difference between MCI and AD was in the *Z*-score of these two regions. The above trend was also observed in the multiple comparison tests of the three groups in Mask(−).

**Table 5:** Summary and comparisons of mean *Z*-scores and atrophy correction rates for the five dementia-related regions from Tables 2, 3 and 4

	Mask(−)			Mask(+)			Atrophy correction rate (%)
	Normal	MCI	AD	Normal	MCI	AD	Mean (SD)
Superior parietal lobule	1.12 (0.30)	1.97 (0.75)**	1.73 (0.47)*	0.97 (0.26)	1.73 (0.65)**	1.48 (0.43)*	13.2 (1.2)^ф^
Angular gyrus	1.12 (0.34)	1.25 (0.48)	1.98 (0.74)**^§§^	0.93 (0.32)	1.09 (0.43)	1.80 (0.76)**^§§^	13.4 (3.6)^ф^
Supramarginal gyrus	0.99 (0.27)	1.13 (0.38)	1.57 (0.63)**^§§^	0.80 (0.24)	1.02 (0.35)	1.43 (0.60)**^§§^	12.9 (5.4)
Precuneus	1.03 (0.28)	1.76 (0.58)**	1.56 (0.37)**	0.89 (0.22)	1.55 (0.49)**	1.37 (0.35)**	11.8 (0.3)
Posterior cingulate gyrus	0.78 (0.29)	1.68 (0.42)**	1.59 (0.35)**	0.70 (0.24)	1.60 (0.69)**	1.51 (0.58)**	7.0 (1.8)
Average of 5 regions	1.01 (0.17)	1.56 (0.42)**	1.69 (0.35)**	0.86 (0.17)	1.40 (0.38)**	1.52 (0.37)**	11.7 (2.1)

*Z*-score, mean (SD) and atrophy correction rate (%) are shown. Statistical significance of multiple comparison tests is expressed as* and ^ф^ for *p* < 0.05, and ** and ^§§^ for *p* < 0.01. Mask (−): without the masking procedure. Mask (+): with the masking procedure

*Normal* cognitively normal; *MCI* mild cognitive impairment; *AD* Alzheimer’s disease

Again, multiple comparison tests of the mean atrophy correction rates revealed that the values of the superior parietal lobule and the angular gyrus were significantly greater than those of the other three regions (*p* < 0.05). In contrast, the atrophy correction rate of the posterior cingulate cortex in each of the three groups was less than 10%, i.e., mean (SD), 7.0 (1.8), suggesting that the posterior cingulate cortex was less susceptible to cerebral atrophy.

**Fig. 4:**
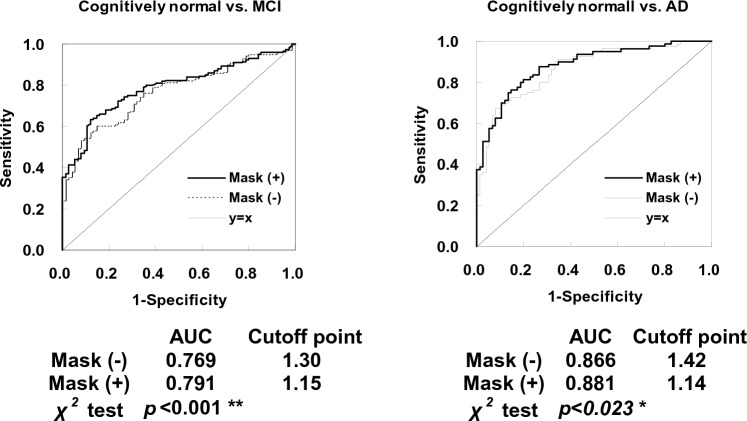
Receiver operating characteristic (ROC) curve analysis of the *Z*-scores Mask (−) and Mask (+) in the five dementia-related regions. In cognitively normal versus MCI and cognitively normal versus AD, the AUC was significantly greater for Mask (+) than for Mask (−), respectively, indicating the superiority of Mask (+). *ACU* area under the curve

The results of the ROC curve analysis for five dementia-related regions are shown in Fig. 4. The *Z*-scores of a total 355 ROIs were analyzed. In cognitively normal versus MCI, and cognitively normal versus AD, the AUC (area under the curve) was significantly greater for Mask (+) than for Mask (−) (*p* < 0.001 and *p* < 0.05, respectively), indicating that Mask (+) was more accurate. The diagnostic accuracy of MCI relative to cognitively normal was 67% for Mask (−) and 69% for Mask (+), and that of AD was 78% for Mask (−) and 82% for Mask (+), indicating improved diagnostic accuracy for Mask (+). In MCI versus AD, however, no significant difference of the AUC was observed.

## Discussion

We proposed a novel atrophy correcting diagnostic approach to precisely assess *Z *-scores reflecting rCBF, by minimizing artifacts as well as over- or underestimation caused by cerebral atrophy, thus improving diagnostic accuracy. As the convexity of the frontal and parietal lobes and the posterior medial cerebrum are particularly susceptible to cerebral atrophy in MCI and AD, the Mask (−) *Z*-score obtained by conventional SPM analysis tends to be overestimated. In contrast, the *Z*-scores in the posterior cingulate cortex and the cerebellum tend to be less susceptible to cerebral atrophy.

Application of the method in the present study significantly reduced *Z*-scores and percent extent for most regions, thereby improving diagnostic accuracy in the ROC curve analysis. The difference in AUC seems small, which is due to the fact that the gold standard for diagnosis was based on clinical symptoms and neuropsychological testing. It is assumed that the accumulation of amyloid beta and tau imaging as criteria for further testing would provide more definitive results, which may indicate that the differential diagnosis between AD and MCI is not easy, and further investigation is recommended.

The proposed approach has two main features. First, the approach is semi-automatic and the program is a single package, which makes it faster and more useful in clinical applications. Matlab is required to run the SPM, however, it is already installed in the program, thus a new Matlab is no longer necessary. The proposed method requires brain MR-T1WI with thin slice thickness that allows sufficient segmentation. In Japan, facilities with SPECT systems are generally equipped with MR systems, and imaging information is linked via PACS. In dementia-related diseases, morphological information from cerebral MR is indispensable for accurate diagnosis, and the method to evaluate cerebral atrophy have been widely used. The proposed approach is able to be used by applying the MR images; therefore, no additional MR imaging is required. Since nuclear medicine and radiology departments are in the same organization in Japan, it is easy to incorporate the proposed method into daily medical practice.

Second, this approach provides visual information about the parietal association cortex that consists of the superior parietal lobule and inferior parietal lobule, separating the two. We applied the OM line instead of the AC–PC line as the baseline of the anatomical standardized SPECT and MRI templates. This is because when the OM line is used, the central sulcus is centered on the surface and axial SPECT images, with the frontal and parietal lobes clearly distinguished, as well as the superior parietal lobule and the inferior parietal lobule. Since it is extremely important to assess regional CBF in the parietal lobe in the diagnosis of dementia, this procedure allows an accurate evaluation of hypoperfusion in the parietal lobe. Furthermore, the anatomically standardized templates are symmetrical, allowing for comparison of right–left differences in *Z*-scores and percent extent values for the target patient.

In an examination of five dementia-related regions, it was found that the parietal association cortex had large atrophy correction rates, and the *Z*-scores Mask (−) were significantly affected by cerebral atrophy. As assessment of hypoperfusion in the parietal association cortex is important, it is necessary to consider overestimation due to cerebral atrophy. In this sense, the approach presented is useful.

Furthermore, higher *Z*-score, i.e. decreased rCBF, in the angular gyrus and the supramarginal gyrus: the inferior parietal lobule may be a finding that distinguishes AD from MCI with mask or without mask procedure. Multiple comparison tests showed significantly higher average *Z*-score in MCI and AD compared to cognitively normal, which is consistent with previous reports. However, when focusing on the inferior parietal lobule, the *Z*-score in MCI was comparable to cognitively normal, while it was significantly higher in AD, indicating a significant difference between the two. Hirao et al. [22], in an interesting study on the conversion from MCI to AD, has reported that rCBF reductions in the inferior parietal lobule and precuneus has high predictive value, although their study was not corrected for brain atrophy, which is consistent with our findings. The details of these findings require further study.

On the other hand, the *Z*-score in the posterior cingulate cortex tends to be less susceptible to cerebral atrophy than other regions; therefore, the value of which may adequately reflect rCBF in cognitively normal, MCI, and even AD. Matsuda et al. [23] reported a greater decrease in rCBF than gray matter volume in the posterior cingulate cortex of mild AD, meaning less atrophy, which is consistent with our present findings.

## Conclusion

1. The proposed atrophy correction method enables appropriate *Z*-score assessment with reduced artifacts caused by cerebral atrophy.
2. The *Z*-scores of the convexities of the frontal and parietal lobes and the posterior medial cerebrum, especially the superior parietal lobule and angular gyrus, are susceptible to cerebral atrophy.
3. The effect of cerebral atrophy on overestimating the *Z*-score is less pronounced in the posterior cingulate cortex and the cerebellum.
4. Higher *Z*-score, i.e. decreased rCBF, in the inferior parietal lobule may be a finding that distinguishes AD from MCI.

### Limitations

Although SPM2 was used throughout the proposed approach, which may be integrated into the program, segmentation was not perfect, and some artifacts still remained. Higher versions of SPM or DARTEL are recommended for future studies. Brain MR-T1W imaging is essential to execute the proposed method; if MRI is not available, brain CT is being devised as a substitute.
